# Menstrual health and hygiene knowledge among post menarche adolescent school girls in urban and rural Tanzania

**DOI:** 10.1371/journal.pone.0284072

**Published:** 2024-03-11

**Authors:** Robert M. Njee, Calister P. Imeda, Said M. Ali, Adiel K. Mushi, Doris D. Mbata, Albert W. Kapala, Emmanuel A. Makundi, Vitus A. Nyigo, Albert M. Majura, Winfrida O. Akyoo, Yolanda J. Mbatia, Germana T. Baraka, Judith M. Msovela, Ester S. Ngadaya, Mbazi F. Senkoro, Hamisi M. Malebo

**Affiliations:** 1 National Institute for Medical Research (NIMR), Dar es Salam, Tanzania; 2 Public Health Laboratory Ivo de Carneri (PHL-IdC), Wawi, Chake, Pemba, Tanzania; 3 National Bureau of statistics, Dodoma, Tanzania; 4 Independent Researcher, Dar Es Salaam, Tanzania; 5 Muhimbili University of Health and Allied Sciences, Dar Es Salaam, Tanzania; 6 St. John’s University of Tanzania, Dodoma, Tanzania

## Abstract

Adolescent girls’ capacity to lead healthy lives and perform well in school has been hampered by their lack of awareness about menstruation and the requirements for its hygienic management. Lack of enabling infrastructure, improper menstrual supplies, and limited socioeconomic support for good menstrual health and cleanliness are characteristics of schools in Africa South of the Sahara. We evaluated school-age girls’ knowledge of menstrual hygiene and identified bottlenecks that could affect policy and programming for menstrual health and hygiene. A school-based cross-sectional study involved 8,012 adolescent school girls in the age group of 11–18 years (mean age = 14.9 years). The study evaluated students’ knowledge of menstrual health and hygiene (MHH) from the viewpoints of schools and communities using a combination of qualitative and quantitative approaches. Data was collected using self-administered surveys, focus group discussions, in-depth interviews, and site observations. Girls’ older age (AOR = 1.62, P 0.001), having a female guardian (AOR = 1.39: P = 001), and having a parent in a formal job (AOR = 1.03: P 0.023) were positively associated with Menstrual health and Hygiene Knowledge. MHH knowledge levels varied significantly between girls attending government (53.3) and non-government schools (50.5%, P = 0.0001), although they were comparable for girls attending rural and urban schools. Only 21% of the study’s schools had at least one instructor who had received training in MHH instruction for students. We have established that the majority of adolescent girls in schools have inadequate knowledge on menstrual health and hygiene, and that school teachers lack the skills to prepare and support young adolescents as they transition into puberty. Concerted actions aimed at building supportive policy are paramount, for school-aged teenagers to learn about and reap the long-term advantages of good menstrual health practices.

## Background

Good hygiene during menstruation is critical in maintaining health and wellbeing [1]. However millions of women and girls suffer from menstruation related infections for lack of knowledge [2–5]. Adolescents in low and middle income countries encounter significant difficulties navigating through menarche because they are largely uninformed and unprepared [6,7].

Menarche is the most prominent event in puberty. It is also associated with characteristic physical and social changes that increase demands for body maintenance and copying with transitioning socio identity [8,9]. In addition to the knowledge gap, menstrual health needs are not supported by the physical or social environments in schools [10]. Several reports cite girls’ challenges with and competition for resources for bathing, washing, and absorbents while in school [11]. Girls’ capacity to pursue education is hindered by unmet requirements for basic menstrual health and hygiene and knowledge of pubertal changes, which could have long-lasting effects on their health and socioeconomic development.

Provision of adequate menstrual health and hygiene (MHH) in schools is paramount for healthy development and learning. Adequate MHH is defined elsewhere encompassing provision for absorbent materials, soap and water, supportive infrastructure, correct and timely knowledge, psychological and socio support [7,8,10,12,13]. Adequate MHH services in school involves two major pillars namely; socio context (which combines puberty education and awareness, supportive socio atmosphere, and positive behavioral expectations or socio norm) and resource capacity; including access to absorbents, hygiene supplies, and supportive physical infrastructure for sanitation [7,13,14]. The balance in state of the two pillars shapes the adolescent girl’s menstrual experience i.e. hygienic management practices, positive perceptions, and effective containment of menstrual flow [13]. The lack of which contributes to negative impacts on women’s and girls’ lives through harm to physical and psychological health, education and social engagement [13,15]. Pubescent education is particularly delicate in the socio context. Teenage girls’ capacity to seek education and make informed decisions about their sexuality and reproduction has been demonstrated to be directly impacted by their lack of pubescent knowledge [16,17].

In Sub Sahara Africa, the onset of puberty among girls coincides with girls beginning to drop out of school, report shows. Additionally, evidence suggests that school absenteeism may be related to menstruation, particularly when MHH in schools is insufficient [18]. However, there is a dearth of work examining MHH knowledge gap among adolescent girls and boys.

Timely and correct MHH knowledge builds upon girls capacity to effectively master the menstrual and other pubertal changes through diverse socio and resource constraints [11,19]. According to a study conducted in Ghana, education for girls increases school attendance and overall academic achievement [16]. Knowledge also affects girls’ attitudes toward healthy behaviors, making it a crucial factor in determining girls’ menstrual health [13]. Menstruation myths and unfavorable attitudes contribute to low self-image for girls and women, according to a study on the needs of girls in camps for refugees [20].

Another important study shows that lack of knowledge is linked to boys’ low self-esteem and unhealthy behaviors as well as girls’ impaired capacity to manage menstruation, stress, humiliation, embarrassment, perplexity, and fear [17,21–23]. It is also reported that educating boys reduces stigmatizing since it prevents misconducts that leads to teasing and shame.

Limited adolescent girls and boys knowledge is a significant challenge that remains to be addressed in sub-Sahara Africa [24,25]. However, there is limited research addressing MHH knowledge, and those that are available are often of a limited scale. We present findings of a large study on adolescent schoolgirls’ knowledge on menstrual health and hygiene management across Tanzania. One of the first attempts to categorize menstrual hygiene knowledge into themes that are pertinent to policy and planning is presented in this article.

## Methods

### Study design and settings

This was a cross-sectional study using a mix of qualitative and quantitative methods for data collection in order to comprehensively compile the views of school girls on Menstrual Health and Hygiene (MHH). This school based study was designed to assess Knowledge Attitude and Practices regarding MHH among school girls. Participants (mainly primary and secondary schools girls) were selected purposively to represent socio cultural and geographical diversity in Tanzania [26]. Nineteen administrative regions were selected namely: Tanga, Dar es Salaam, North Unguja, and North Pemba, in Coastal Zone; Arusha and Kilimanjaro, in Northern Zone; Shinyanga, Kagera, and Mara in Lake Zone; Dodoma in Central Zone; Kigoma and Tabora in Western Zone; Iringa and Mbeya in Southern Highland Zone; and Mtwara and Ruvuma in Southern Zone. Study districts and study schools, were randomly selected using a combination of bowl lottery and the probability of proportional to size (PPS) respectively [27].

### Sample size and recruitment

Eligible participants were upper primary school girls (grade five to seven) and all secondary school girls. Post menarche girls were selected purposively with the help of matron or teacher responsible for girls’ welfare. We aimed at enrolling 9,600girls from 16 districts, and 320 schools. Parallel groups of 12 post-menarche girls and 12 adolescent boys per school were invited to separate Focus Group Discussions (FGD).

Secondary participants included school matrons and teachers, and key informants comprising of members of administrative units and partner organizations at district and national levels, and representatives from communities that own the schools. Exclusion criteria for school girls were pre-menarche status and inability to participate in interviews during data collection.

### Data collection

Quantitative data was collected using self-administered questionnaires. Qualitative information was collected through FGDs, In-depth Interviews (IDI)s and Key Informants’ Interviews (KII)s using interview guides.

### Quantitative data analysis

Data was processed using Census and Survey Processing System 7.2 (CSPro 7.2) and STATA 15. Descriptive analysis including frequencies, percentages and measures for the central tendency, student t-test were employed. Significance of differences and association was tested at 5% level. We broke down the knowledge into six sub themes independently i.e. “the meaning of menarche (menarche)”, “understanding of the term menstruation (Menstruation)”, “identifying signs that menstruation period is about to start (signs for start)”, “what actions should a girl take if she experience onset of menstruation while in class (class incidence)”, “preparatory actions advised in case of approaching menstrual period (preparations)”, and “correct use of menstrual materials (use of materials)” in order to explore the type of knowledge gap for this study population.

#### Dependent variables

Knowledge was determined as percentage scores from correct responses out of total scores allocated in the knowledge items. Each of the six knowledge questions (addressing knowledge sub theme) was allocated equal weight regardless of the number of correct responses. Participants were considered as having adequate knowledge of MHH if they had a mean percentage score of 70% or more. We adopted this cut of point to be comparable with a similar study from sub Saharan Africa [27].

#### Independent variables

The just aforementioned factors were associated with socio-economic and demographic factors/variables. These variables were extracted from girls’ questionnaires.

#### Modeling

Logistic Regression analysis and reported both unadjusted and adjusted odds ratios with 95% confidence intervals was used to test associations between knowledge and socio demographic determinants.

### Qualitative data analysis

Qualitative data collection methods complemented the quantitative component allowing participants to provide insights to their experiences. School children participated through Focus Group Discussions (FGDs); parents, guardians, and cultural leaders took part in FGD or In-depth Interviews (IDIS); teachers, administrators, and stakeholders responded to Key Informant Interviews (KII) accordingly. The qualitative approach was built on Grounded Theory [28,29]. Data was manually analysed with coding of verbatim transcripts from IDIs, KIIs and FGDs. The coding process led to obtaining categories which were directly derived from the text data in line with qualitative content and thematic analysis approaches [30,31]. This process was carried out by two experienced qualitative data analysts. In turn, two social scientists initially designed the interview and discussion guides independently reviewed the derived categories of text to obtain sub-themes and themes presented in this manuscript.

Data validity was integrated in this study from its design stage to ensure ‘trustworthiness’ of the qualitative findings. Validity was also assured through training of data collectors and during analysis. The strategies employed included day-to-day debriefing sessions and exchange of transcripts, coded texts, and themes among research team members to reduce bias. Agreement and disagreements on the themes and sub-themes were resolved through consensus building among team members. Data presentation is hence backed by verbatim descriptions of participants’ accounts. Data triangulation complimented the strategy by ensuring the examination of data collected from different tools [31,32]. In this paper we synchronize data presentation between quantitative and qualitative approaches to effectively triangulate the information.

Audio files from field were transcribed verbatim to generate transcripts. The transcripts were subsequently coded in line with qualitative content analysis approach where project themes were determined based on study objectives. A member check approach was used to establish trustworthiness of the coding scheme [33]. The independent coders evaluated the list of developed sub-code descriptions and examples for each main code (theme). Further thematic analysis proceeded with description of similarities and differences of observations between districts as the unit of measurements. Each thematic content analysis was accompanied by extraction of supporting quotes and drawing of conclusions and recommendations. However, presentation of qualitative component in this paper was organized to triangulate with quantitative findings.

### Ethical considerations

Ethical Clearance was obtained from the National Health Research Ethics Review Committee of Tanzania. Thereafter, administrative approval was sought from the ministry responsible. Written informed consents were sought from adult participants while school adolescents were given assent forms. Consenting and assenting for and among adolescents was done in the following manner: Researchers introduced the study to eligible groups of adolescents after a head teacher had given a written consent. Adolescents were explained about the research in the absence of the teacher and were invited to sign a written assent. Adolescents were assured of no consequences for not participating and did so in absence of teachers to avoid any sense of obligation.

## Results

### Demographic characteristics of participants and study schools

A total of 294 schools (92% of the target) were enrolled in the study spread throughout 19 districts in both Zanzibar and the mainland of Tanzania. About two-thirds of the schools, or 60.5% (n = 178), were located in rural areas, with the majority (86.4%; n = 254) being owned by the government. A total of 8,012 girls—or 91% of the target population—replied to the self-administered surveys. The limited number of post-menarche girls in schools was the primary factor in the lack of response from girls. The average age of the girls was 14.9 years (SD = 0.02). The average girl’s age at menarche was 12.9 years. Among school adolescent girls 2.5% had physical disability. In comparison to rural schools, private schools, or primary schools, respectively, more students from urban schools (61.3%), public schools (86.0%), and secondary schools (58%) were enrolled in the study. Socio demographic characteristics of the enrolled school girls are summarized in Table 1 below.

**Table 1:** Demographic characteristics of adolescent school girls (n = 8,012).

Frequency distribution		
	n	%
**Age group**	**8,012**	**100.0**
≤13 years	2,030	25.3
>13 years	5,982	74.7
**Parent**	**8,009**	**100.0**
Father	3,828	47.8
Mother	2,504	31.3
Male Guardian	289	3.6
Female Guardian	640	8.0
Other	748	9.3
**Parent’s education level**	**8,012**	**100.0**
Primary	3,313	41.4
Secondary	1,647	20.6
Tertiary	937	11.7
None	874	10.9
Dont know	1,241	15.5
**Parents employment**	**8,009**	**100.0**
Peasant	4,376	54.6
Formal employee	1,266	15.8
Retired	229	2.9
Entrepreneur	1,877	23.4
Dont know	261	3.3

### Girls knowledge levels

The mean overall knowledge score on MHH was 52.9% (52.44–53.42). The difference in knowledge between Government (53.3) and non-government schools (50.5%) was statistically significant (P = 0.0001), whereas knowledge levels for rural (52.5%) and urban schools (53.2%) were comparable. On MHH, the average total knowledge score was 52.9%. (52.44–53.42). While knowledge levels in rural (52.5%) and urban (53.2%) schools were comparable, there was a statistically significant difference in knowledge between government (53.3) and non-government schools (50.5%; P = 0.0001).

Table 2 displays the responses from schoolgirls on knowledge assessment questions. The answers to the six core knowledge assessment questions, each of which represents a knowledge subtheme (hereinafter referred to as a domain), are completely displayed, together with the percentage of correct and incorrect answers for each choice. Along with observations on how participants responded to the right questions, Table 2 also shows how common it is for adolescent schoolgirls to have misconceptions about menstruation. Menstruation being the point of transition from childhood to adulthood (A.4), menstruation lasting the entirety of a woman’s life once it starts (B.1), confusing pubertal changes and menstrual changes (C.1), and the notion that used menstrual devices can be disposed of in any hole or toilet are some of the myths that relatively large proportions of respondents believed to be true (F.2). Girls from rural or urban schools don’t seem to respond differently in any discernible pattern.

**Table 2:** School adolescent girls’ responses to knowledge questions on menstruation and menstrual hygiene (n = 8012), correct responses are highlighted.

S/N	Domain and Item	Rural, n(%)	Urban, n(%)	Total, n(%)
**A**	**What do you think menarche (onset of menstruation) is about?**			
1.	Bleeding from a wound	59 (1.9)	197 (4.0)	256 (3.2)
2.	Bleeding from the genitals without problem	1,987 (64.1)	3,304(67.3)**	5291(66.06)
3.	Getting the first menstrual blood	1,400(45.2)	2,256(46.0)	3,656 (45.65)
4.	When a girl goes from childhood and into adulthood	2,346(75.7)	3,552(72.3)***	5,898 (73.62)
5.	The onset of puberty in girls	1,510(48.8)	2,338(47.6)	3,848 (48.05)
6.	End of virginity	114(3.7)	172(3.5)	286(3.57)
**B**	**In your opinion, which of the following are true about menstruation?**			
1.	When it begins it lasts for the whole life of a girl / woman	1021(33.0)	1,652(33.7)	2673 (33.37)
2.	Menstruation begins in puberty	1447(46.7)	2237(45.5)	3,684 (46.00)
3.	Normal menstruation can cause anemia	275(8.9)	521(10.6)***	796 (9.94)
4.	Menstruation often causes a girl to get sick	1,045(33.7)	1,896(38.6)	2,942 (36.72)
5.	Blood flow usually ranges from 3 to 7 days	2178(70.3)	3523(71.7)	5701(71.2)
6.	Represents active reproduction age	1051(33.9)	1907(38.8)***	2958(36.9)
**C**	**Which of the following commonly occur before the beginning of menstrual flow?**			
1.	Rapid growth, weight gain, and soft voice	2,283(73.7)	3,732(75.4)	6,015 (75.10)
2.	Emotional changes, irritable mood, soft / enlargement of breasts	2,089(67.4)	3,282(66.8)	7,371 (67.06)
3.	Loss of appetite, coughing, sneezing and nausea	240(7.7)	592(12.1)	832 (10.39)
**D**	**Which of the following is correct action that girl/woman should take when she knows she is about to start her periods?**			
1.	Changing her school schedules and work	219(7.0)	399(8.1)**	618 (7.72)
2.	Make sure she has menstrual supplies in her bag	2,278(73.5)	3,464(70.6)*	5,742(71.69)
3.	Buy special fabrics and packaging	1,367(44.1)	2,408(49.0)***	3,775 (47.13)
4.	Avoid gatherings, games and socializing	530(17.1)	879(17.9)	1,409 (17.19)
5.	To send all her school items home	109(3.5)	242(5.0)***	354 (4.42)
6.	Preparing a clean and confidential place for exchange	2,201(71.0)	3,910(74.5)**	5,860 (73.17)
**E**	**Which of the following are advised if a girl is in class and feels that her period is about to start?**			
1.	Go home very quickly	501(16.2)	938(19.1)	1,439 (17.97)
2.	Tell friends about her condition	591(19.1)	1,204(24.5)	1,795 (22.41)
3.	Ask for supplies from teacher or matron	1,938(62.5)	2,807(57.2)	4,745 (59.25)
4.	Ask for permission first before going home	1,062(34.3)	1,976(40.2)	3,038 (37.93)
5.	Going into special rooms for needs	1,424(46.0)	2,331(47.5)	3,755 (46.88)
6.	Go to the toilet and put on a pad or towel	1,743(56.2)	2,899(59.0)**	4,642 (57.96)
**F**	**Which of the following is true concerning the use of menstrual materials?**			
1.	The menstrual devices should be replaced after 24 hours	989(31.9)	1,584(32.3)	2,573 (32.13)
2.	Used menstrual devices can be placed in any hole or toilet	1,480(47.8)	2,644(53.9)	4,124 (51.49)
3.	Sun rays and wind helps prevent infections in cleaned pads	435(14.0)	783(16.0)	1,218 (15.21)
4.	Only industrial menstrual materials should be recommended for use by school girls	808(26.1)	1,481(30.2)	2,289 (28.58)
5.	Using unclean menstrual devices can lead to infections	1,704(55.0)	2,657(54.1)	4,361 (54.45)
6.	Menstrual materials can be shared between friends	173(5.6)	331(6.7)	504 (6.29)

### Menstrual hygiene knowledge domains

**Fig 1:**
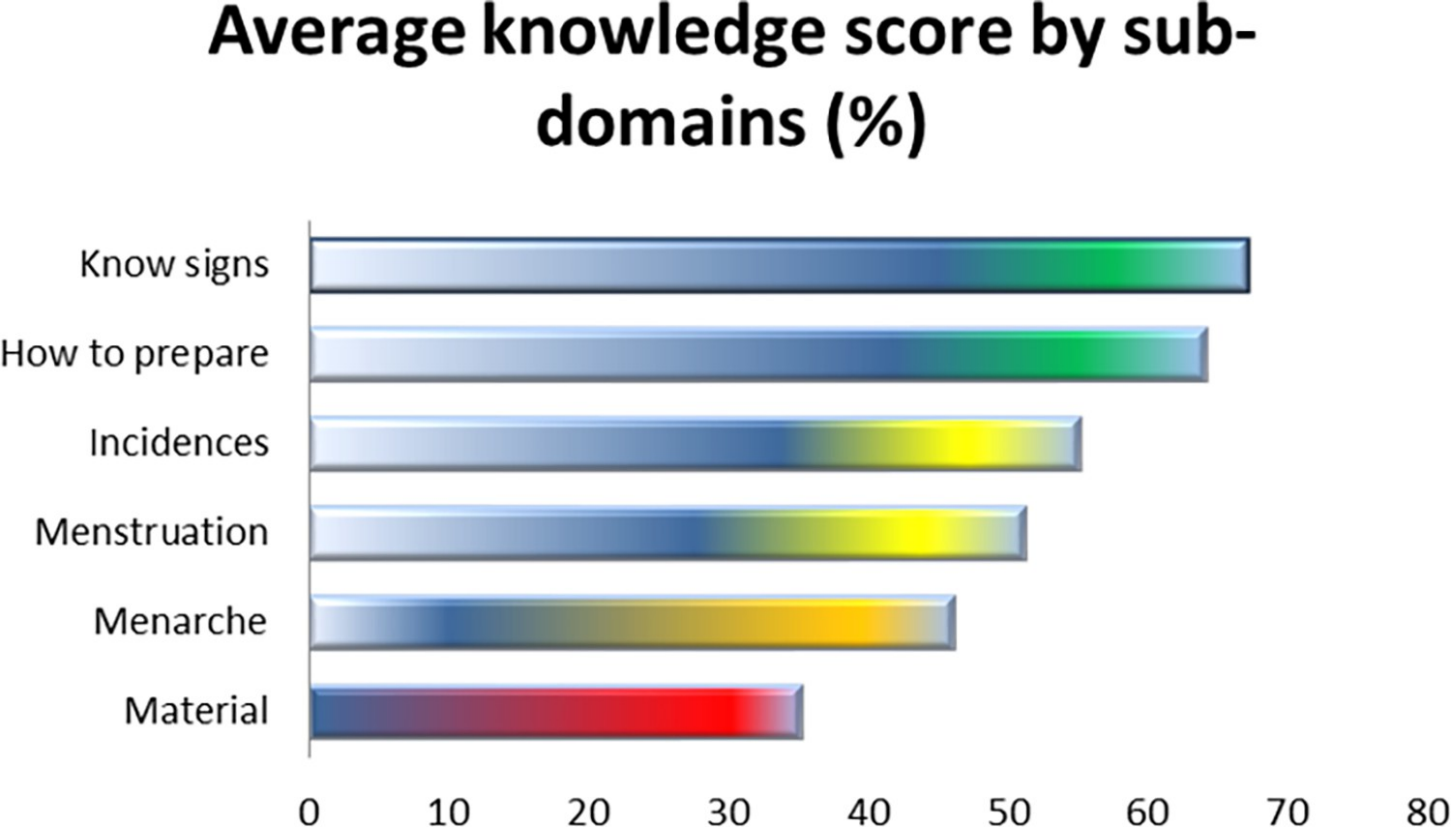
Average girls’ knowledge on specific menstrual hygiene management domains MHH Knowledge adequacy.

*Fig 1* Display the performance percentage of girls in each of the six knowledge domains. Average girls’ knowledge differed significantly between domains, with information about "signs that menstruation was about to start" scoring the highest and knowledge regarding "proper usage of menstrual products" scoring the lowest.

When we applied 70% as the cut-off for knowledge adequacy, we observed that 24% of the girls (22.96–24.83) had adequate knowledge on MHH. Compared to the lowest age group, the proportion of people in the oldest age group who possessed adequate knowledge was more than two folds higher. The proportion of secondary school girls with adequate knowledge was higher, and the difference was statistically significant (P 0.05). There was no statistically significant difference in knowledge adequacy between girls in government-run and privately-run schools, as well as between those from rural and urban areas.

The responses obtained through FGDs from adolescent boys and girls provide us with more details about their knowledge of MHH. In this instance, responses from boys are provided to put post-menarche girls (who experienced periods) in comparison to their peer adolescent boys who might learn differently or may have unique perspectives on the subject. The quotes below show the typical responses by post-menarche girls and adolescent boys as they described menstruation.

*“Is a change that happens to a girl in her body; that is*, *blood would come off every month”* (FGD boys, Karatu District).

*“…menstruation is the transition from childhood to adult hood”* (FGD girls, North Pemba District).

Some of the girls and boys were more explicit in describing the concept of menstruation and gave out descriptions like;

*“…it constitutes maturing of an ovum in a woman and failure of fertilization by a male seed so that it is released from the body through the vagina”* (FGD girls, Temeke District).

*“*..*the signs of menstruation are such that blood comes from girl’s private parts*, *I heard that*…*”* (FGD boys, Rorya District).

Boys’ description of menstruation revealed their own perception of the process that might be a form of misconception. Below we present quotations from FGDs that highlights some of the perspective brought out from boys as they explained the meaning of menstruation;

*“…it is a circle woman get monthly and it may occur once or twice a month according to the changes; it allows discharge of dirty blood from her private parts”*(FGD. Boys, North Pemba Region).

*“Menstruation is the period when ovum of girls/women discharged from the ovary in the form of dirty mucus that put women in two periods*, *safe and unsafe period for getting pregnant”* (FGD Boys, Mpwapwa District).

*“Menstruation is the release of ovum from ovary and later gets excreted as dirt”* (FGD Boys, Mbeya District).

Boys frequently used the word "dirt" when speaking about their periods, which may show how they view the process or the results. Additionally, several of the descriptions from both girls and boys suggested common misunderstandings regarding the menstrual cycle, particularly as it relates to fertility. The misconceptions surrounding the menstrual cycle and the possibility of pregnancy are presented in the quotes below;

“Menstruation is where a woman is in her period if she engages in sex, she may get pregnant because the ovum will have left from ovary and move to a place where if you get sexually involved with her she will get pregnant” (FGD boys, Temeke District)

“I normally talk to my mom and my sister, they often tell me when I am in my period I should keep away from boys since they can make me pregnant and cause me to lose my dreams” (FGD girls, Mbeya).

Another important viewpoint that was expressed is the one about the connection between menarche, menstruation, and maturity. Individuals had different maturity perspectives towards menstruation, and FGDs and IDIs also revealed this variability. The quotes that follow reflect some of the prevalent conceptions and myths about menarche as a sign of maturity. One of the very common

*“Menstruation is the last stage of growth from childhood into adulthood”* (FGD, Girls, Karatu District).

*“The community considers the issue of menstruation such that when a girl reaches her menarche*, *she is already grown up*, *ready for marriage; they would even hold her from pursuing her studies”* (IDI. Matron, Igunga District).

### Factors associated with menstrual hygiene knowledge

A logistics regression analysis illustrates the relationship between participants’ socio economic characteristics and knowledge adequacy. Being older than 13 years old, having a mother and a female guardian, and having a parent in a formal job were positively and significantly associated with knowledge adequacy (P < 0.05). However, parents’ education was not associated with girls’ knowledge adequacy on MHH (Table 3).

**Table 3:** Logistic regression outputs summarizing associations between knowledge adequacy and selected demographic variables.

Covariate	Multivariate analysis (n = 8,009)			
	OR Adjusted	95% Confidence Interval (CI)		p-value
		Lower limit	Upper limit	
**Age group**				
≤13 years	1.00	—	—	—
>13 years	1.62	1.42	1.84	**<0.001**
**Parent**				
Father	1.00	—	—	—
Mother	1.13	1.00	1.27	**0.044**
Male Guardian	1.16	0.88	1.53	0.280
Female Guardian	1.39	1.15	1.68	**0.001**
Other	0.98	0.81	1.18	0.828
**Parent’s education level**				
Primary	1.00	—	—	—
Secondary	0.97	0.84	1.12	0.697
Tertiary	0.85	0.69	1.03	0.105
None	1.02	0.86	1.21	0.813
Dont know	0.94	0.80	1.10	0.411
**Parents employment**				
Peasant	1.00	—	—	—
Formal employee	1.21	1.03	1.43	**0.023**
Retired	1.17	0.85	1.60	0.343
Entrepreneur	1.03	0.90	1.18	0.658
Don’t know	0.79	0.57	1.09	0.154

#### Sources of information, timing and type of instructions about menstruation

**Fig 2:**
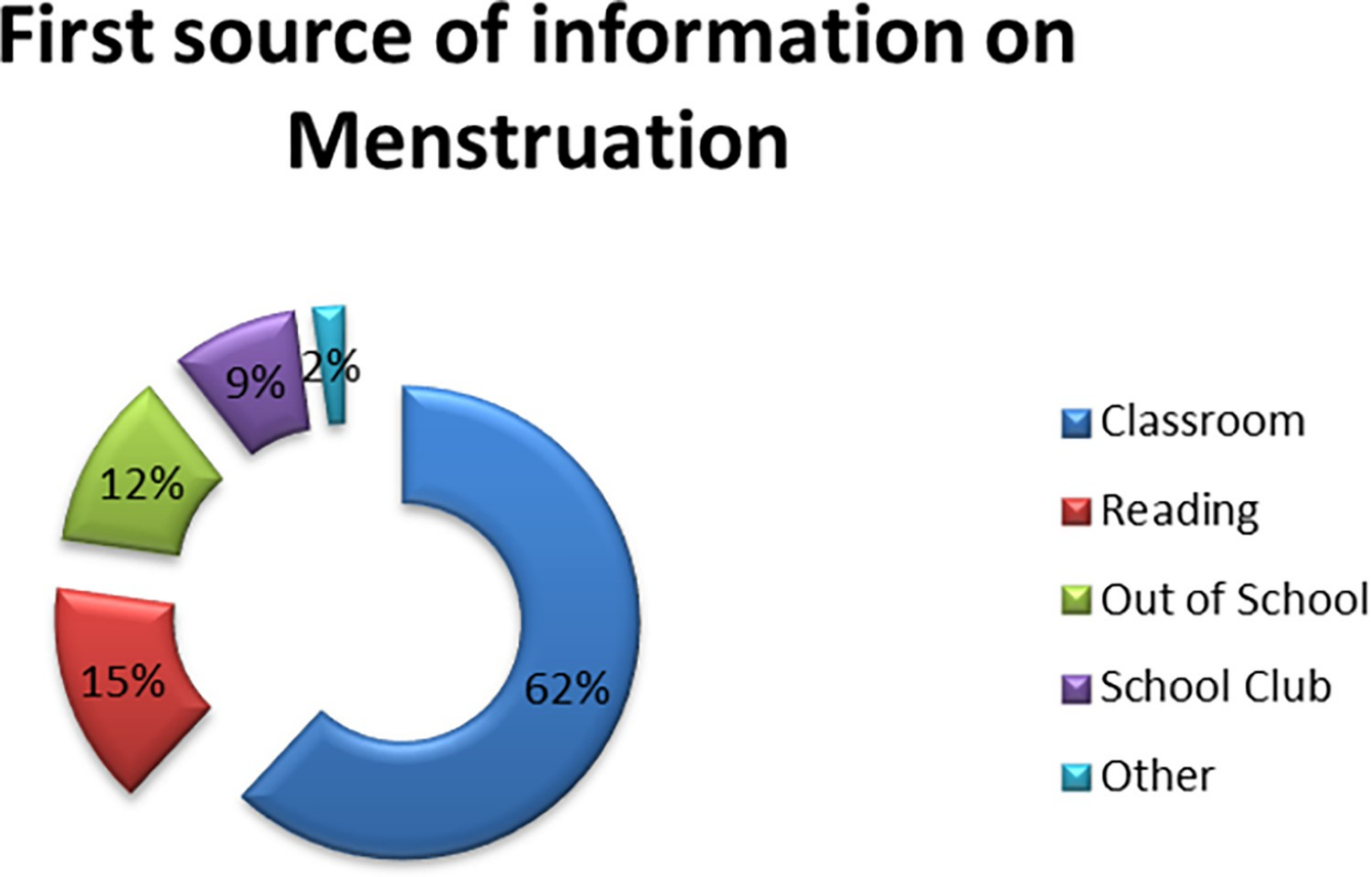
Girls’ first-ever sources of menstrual health information.

**Fig 3:**
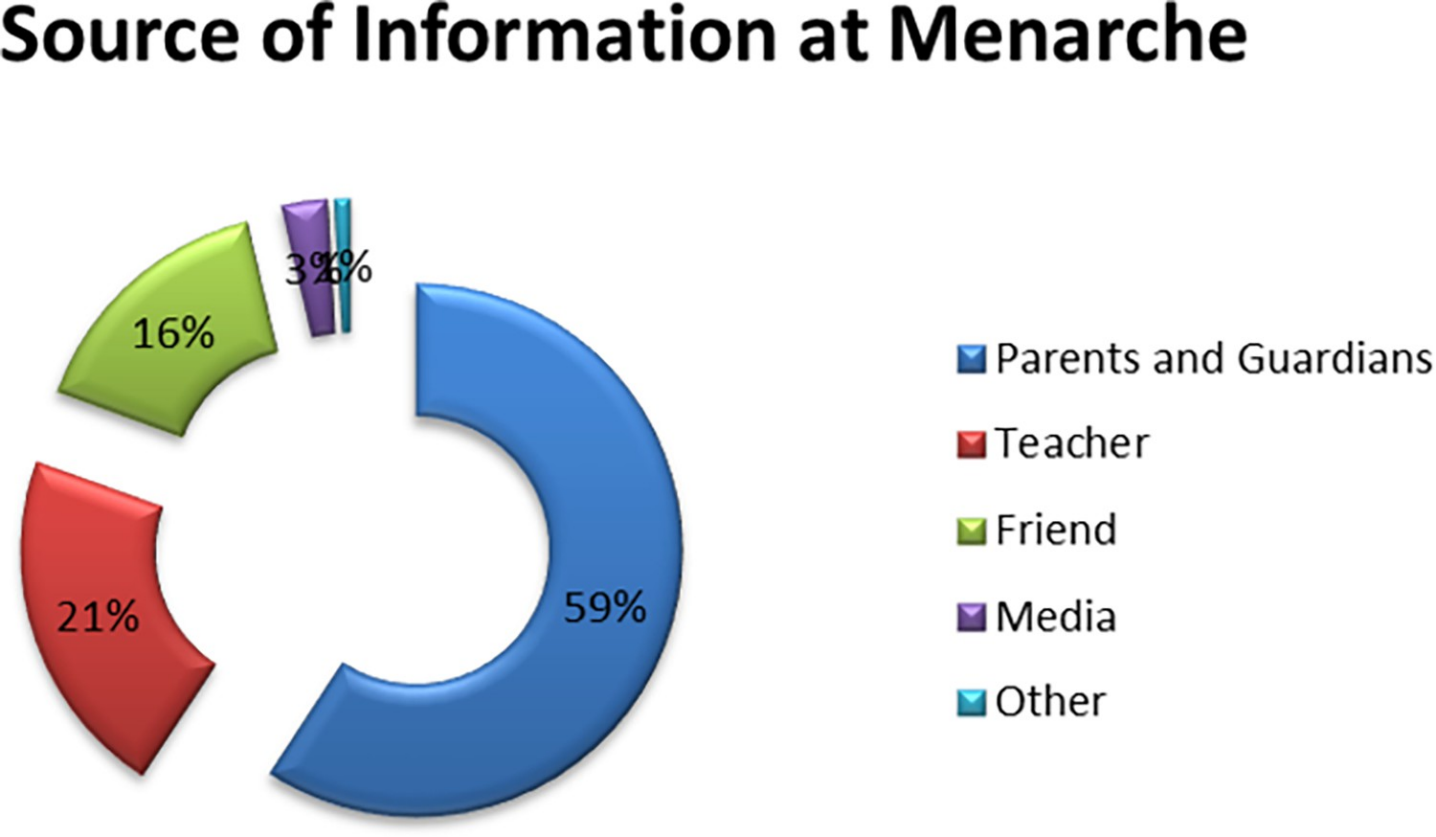
Source of information on menstrual hygiene materials at menarche.

For the majority of respondents (70.5%), learning about menstruation first came through their school setting, with many hearing about it mostly in class sessions. This suggested that among school-aged adolescent girls, the most effective place to learn about MHH was at school (See Fig 2). On the contrary, over two thirds of the girls who were asked where they learnt about menstrual hygiene in detail said they did it from their parents. Only around one in five students in schools had a detailed introduction to MHH. Those who acquired the information at school expressly named teachers as the source rather than the class sessions as shown in Fig 3.

Respondents were asked if they learned about menstruation before or after menarche in addition to the information source. By the time they reached menarche, the majority of the girls claimed to have heard of menstruation, but 30% hadn’t till menarche. In those who had never heard before menarche, 54% did so during the actual menarche, while others were not instructed until after they had dealt with it. Compared to government schools (67.4%), more pupils from non-governmental schools (75.6%) said they had heard about menstruation before menarche.

Qualitative interviews offer further insight about girls’ knowledge of menstruation when they reached menarche. Some of the girls reported to have been utterly oblivious of the changes occurring in their bodies when asked to describe their experience, while others claimed to have known what it was and even what they needed to do to deal with the issue. The quotes that follow list the girl participants’ experiences that are relevant to their understanding of the incident;

*“When I saw myself bleeding I screamed*, *my grandmother asked me why you are screaming*, *don’t be afraid*. *She made cuts of cloths and showed me how to use them; later in the evening she bought sanitary pads for use the day after in school”* (FGD, Girls, Karatu District).

*I was in my room watching TV*, *I saw blood*, *I told my aunt*, *my aunt told me to show it to my mother*, *my mother said I do not want to see it*, *go talk to your grandmother*. *“Grandmother told me to pick a pad from cupboard and wear it… later she told me you have grown up”* (FGD girls, Lushoto District).

A few girls, demonstrated confidence and awareness about correct actions required for them to manage menstruation. As expressed in this quote from a post menarche adolescent girl below;

*“I was not worried at all*, *I use to live with my aunt at that time*… *I did not tell her until 4 months had passed (because my mother had travelled)*, *and I knew what had happened so I knew how to take care of myself*. *I went and bought sanitary pads myself and wore it”* (FGD, Girls Temeke District).

We observed that just 21% of schools had at least one teacher who had received training in MHH instruction for school-aged children. In comparison to their respective urban schools (15.5%) and government owned schools (19.7%), rural schools (24.7%) and non-government owned schools (30.0%) had slightly greater compositions of trained teachers. Although there was a revised curriculum and textbooks on the subject, 94.9% of matrons and school heads in the study schools were unaware of the existence of any specific teaching material or guide for teaching MHH. The following quotations from key informants and school teachers who participated in interviews attest to the shortage of qualified instructors and mentors.

*“I deliver that knowledge as a mother*, *a parent*, *and as a teacher and guardian… the knowledge I have is what I give to the children*, *however I do not have formal guideline*, *neither have I taken part in any seminar concerning menstruation”* (IDI, Matron, Temeke District).

*“But it is very difficult for us to teach this because in class we have both males and female kids*, *so you cannot teach in-depth”* (FGD—CORPs, Teacher, Mbeya).

The timing of adolescents’ MHH education is also related to their level of knowledge. As can be seen in the quotes below, during the focus group discussion with the community, they clearly asserted that the ideal moment to inform teenagers about MHH concerns is when they have already begun menstruating.

*“When a child reaches that time of maturity*, *that is when you can teach her and explain to her the negatives*, *but before she reaches that stage it is like you are initiating bad habits on her*. *A good example is pregnancy*, *someone would have to be pregnant first before you can explain to her when a child wants to move in her belly is like this*, *and how you know when you are about to give birth*, *things like that*. *You have to wait until a girl reaches menarche”* (FGD CORPs, Urban).

*“In the community that I live of Bena*, *once a girl has reached menarche*, *that is the time you arrange a family meeting and she’d gets to be told about all her bad habits*, *after that she will have to thank everyone in that meeting*. *That is how it is done traditionally”* (FGD CORPs, Mbeya District).

The reason behind doing this was a common belief that teaching a girl before menarche amounts to an error of initiating sexual activity as referred to as “bad habits”.

## Discussion

Adolescent girls’ capacity to handle menstruation and achieve their life potential is constrained by the lack of knowledge. In the present study, we have taken into account the sample size limitations and used mixed methods to thoroughly evaluate the question about Menstrual Health and Hygiene knowledge. Our findings imply that the majority (72%) of school-aged girls lack adequate knowledge of hygienic menstruation management and are therefore ill-equipped to deal with menarche and the rest of their reproductive lives.

We found that 30% of school girls remained unaware about menstruation until menarche. This proportion is two folds higher than what was previously stated for Tanzania [34]. The results, however, are consistent with a substantial body of research from Africa and other Low and Middle Income Countries. Quantitative research from Iran, India, Ethiopia, and South Africa found that, by the time they reached menarche, 27.2, 27.7, 22.2%, and 25.3% of females, respectively, had never heard of menstruation [35–38]. Similar findings from qualitative investigations claim that a sizable proportion of adolescent females are still uninformed of menstruation by the time they have their first period [22,25]. Despite the wider consistency, a few other studies have suggested that awareness levels before menarche may be higher in some locations with those unaware by menarche remaining as low as 3.6% [39,40].

We have shown that the majority of school-age girls receive thorough instruction on menstrual management at menarche, primarily from their immediate family, moms in particular. This finding is in line with a large body of literature from various cultures and ethnicities [6]. Additionally, we have demonstrated in the current research that Tanzanian schoolgirls only receive general awareness training prior to menarche. The amount of information provided is seen to be insufficient to give young adolescents the skills they need to deal with the hygienic management of menstruation when it occurs.

We have identified teaching skills as a critical structural gap in knowledge transfer that hinders educating students for healthy menstruation and a better future. This was possible by combining quantitative and qualitative research methods. For instance, some of the instructors in our study group felt it was inappropriate to discuss menstruation in classes with both boys and girls, while others thought it was better to wait until menarche. Without training, instructors’ attitudes continue to be shaped more by their sociocultural upbringing than by scientific evidence. Albeit, a study from Ghana found that formal teaching competencies are crucial for improving the effectiveness of MHH instruction [41]. Our results are also consistent with observations from previous scholars on this subject. Veleshala *et*. *al*. (2020) raised concern over limited contribution of school teachers in knowledge transfer as reported by girls in India, whereas other findings indicate that instructors in Zambia and Kenya were lacking expertise on the subject [25,28]. However, ours is one of the few initiatives to examine the antecedents of knowledge regarding menstrual hygiene in Africa. In the absence of sufficient technical proficiency and resources for MHH training, it would be expected that classroom instructions would vary depending on the backgrounds of the teachers. Thus, these findings point to both the structural issues and the actual requirement for menstrual health and hygiene education in schools.

When knowledge was broken down into categories, we discovered that around a quarter (24%) of schoolgirls had sufficient knowledge of MHH. These girls were able to respond to questions about several domains of menstrual hygiene management with at least 70% accuracy. Our findings are on par with a few related ones from other areas. While Sivakami et al. (2015) found that around a quarter of Indian girls had some understanding about menstruation, Siabani et al. (2018) found that 32.4% of school-age adolescents in Iran had good or fair knowledge of MHH [19]. However, analogous research conducted in Africa revealed greater levels of knowledge. While Fehintola et al. (2017) showed 55.9 excellent knowledge in Nigeria, two studies from Ethiopia claimed 70% coverage of good knowledge in respective populations, more than double our results [27,35]. Due to methodological differences among studies, direct comparison may not be feasible. Despite this, our study is probably the most reliable given its broadly representative sample size, which is at least an order of magnitude larger than that of most other papers—except in cases where there are significant socioeconomic and structural variations. The findings therefore point to a broad inadequacy of menstrual hygiene knowledge among adolescent schoolgirls. It is arguable that teenagers who do not attend school will likely have knowledge levels that are much lower without formal education. The misunderstandings examined in-depth in the qualitative component are a clear indication of the knowledge gap in this community. The myths we noticed among schoolgirls and the community’s belief system supporting them are well in line with findings from other African countries and beyond [22,25,28].

The greatest performing domains had knowledge adequacy levels that were significantly greater than the lowest, by almost a 2-fold margin. An in-depth analysis of the domains shows that those with higher average performances represented a more practical knowledge type, such as knowledge of menstrual signs, how to prepare for it, and how to handle in expected menstrual incidences. This type of knowledge may be experiential knowledge that is likely to be acquired over time. These findings might help to explain why older girls scored higher on knowledge tests than younger girls. On the other hand, the girls performed less well generally in domains that addressed conceptual questions concerning menstruation, such as what it is or is not. Low conceptual performance may indicate a lack of instruction in fundamental biological concepts related to menstruation; the limitation is expressed in some of the myths found in qualitative data. An excellent indication of adolescents’ lack of knowledge about the menstrual cycle is the fact that both girls and males believe the menstrual period to be a fertile time. Our finding that teenagers lack fundamental knowledge of menstrual biology is consistent with similar research conducted both within and outside of Africa [25,28,42,43]. However, literature argues that it’s crucial to teach females how to choose, care for, and securely discard menstruation materials [28].

## Conclusion

We have confirmed through this study that the majority of school-age girls lack the knowledge and skills necessary to manage their periods hygienically by the time they reach menarche. Although schools continue to play a critical role in preparing adolescents, they often lack the tools and expertise required to convey the knowledge that is so important for this vulnerable population. Our findings also demonstrate that while communities, particularly in this region of Africa, play a significant role in educating girls about menstruation, their influence is limited by entrenched misconceptions and a lack of systematic guidance. MHH knowledge among girls is positively influenced by older age, having a female parent, and parents’ employment. To improve MHH knowledge and associated long-term benefits for school-aged adolescents, concerted initiatives addressing supportive policy and structural change are essential.

## Supporting information

S1 File (CSV)
